# The genetic landscape of immune-competent and HIV lymphoma

**DOI:** 10.1186/1750-9378-7-S1-O1

**Published:** 2012-04-19

**Authors:** Jenny Zhang, Vladimir Grubor, Cassandra L Love, Anjishnu Banerjee, Kristy L Richards, Piotr Miezcowski, Cherie H Dunphy, William WL Choi, Wing-Yan Auv, Gopesh Srivastava, Patricia L Lugar, David A Rizzieri, Anand S Lagoo, Leon Bernal-Mizrachi, Karen P Mann, Christopher R Flowers, Kikkeri N Naresh, Andrew M Evens, Leo I Gordon, Magdalena B Czader, Javed I Gill, Eric D Hsi, Qingquan Liu, Alice Fan, Katherine Walsh, Dereje D Jima, Micah Luftig, Ting Ni, Jun Zhu, Amy Chadburn, Shawn Levy, David B Dunson, Sandeep S Dave

**Affiliations:** 1 Duke Institute for Genome Sciences and Policy, Duke University, Durham, NC, USA; 2 Department of Statistical Science, Duke University, Durham, NC, USA; 3 University of North Carolina, Chapel Hill, NC, USA; 4 The University of Hong Kong, Queen Mary Hospital, Hong Kong, China; 5 Duke University Medical Center, Durham NC, USA; 6 Emory University, Atlanta GA, USA; 7 Imperial College, London, UK; 8 University of Massachusetts, Worcester, MA, USA; 9 Northwestern University, Chicago, IL, USA; 10 Indiana University, Indianapolis, IN, USA; 11 Baylor University Medical Center, Dallas, TX, USA; 12 Cleveland Clinic, Cleveland, OH, USA; 13 Genetics and Development Biology Center, National Heart, Lung, and Blood Institute, National Institutes of Health, Bethesda, MD, USA; 14 Hudson Alpha Institute for Biotechnology, Huntsville, AL, USA

## 

Burkitt lymphoma (BL) and diffuse large B cell lymphoma (DLBCL) are aggressive forms of lymphoma in adults and demonstrate overlapping morphology, immunophenotype and clinical behavior. The risk of developing these tumors increases ten to hundred-fold in the setting of HIV infection. The genetic causes and the role of specific mutations, especially in the setting of HIV, are largely unknown.

The decoding of the human genome and the advent of high-throughput sequencing have provided rich opportunities for the comprehensive identification of the genetic causes of cancer. In order to comprehensively identify genes that are recurrently mutated in immune-competent DLBCL and BL, we obtained a total of 92 cases of DLBCLs and 40 cases of BL. These cases were compared to a set of 5 DLBCLs and BL tumors derived from patients with HIV. The DLBCL cases were divided into a discovery set (N=34) and a prevalence set (N=61). The Burkitt cases were also divided into discovery and prevalence sets (N=15, N=45 respectively). For each of the discovery set cases we also obtained paired normal tissue. We performed whole-exome sequencing for all of these using the Agilent solution-based system of exon capture, which uses RNA baits to target all protein coding genes (CCDS database), as well as ~700 human miRNAs from miRBase (v13). In all, we generated over 6 GB of sequencing data using high throughput sequencing on the Illumina platform.

We identified a total of 432 genes that were recurrently mutated in DLBCL and BL. We found that each tumor had an average of 20 gene alterations, which is fewer than most other solid tumors sequenced to date. Commonly implicated biological processes comprising these genes included signal transduction (e.g. PIK3CD, PDGFRA), immune response (e.g. B2M, CD83, IRF8) and chromatin modification (e.g. MLL3, SETD2). We found that lymphomas that arose in the setting of HIV had fewer mutations overall and had a paucity of mutations related to immune response.

These data implicate the depressed immune response by HIV as a contributing risk factor for the development of lymphomas and suggest that HIV lymphomas are genetically less complex than their immune competent counterparts. This study represents one of the largest applications of exome sequencing in cancer, and provides early clues to the genetic causes of HIV-lymphomas.

